# RNA Interference Restricts Rift Valley Fever Virus in Multiple Insect Systems

**DOI:** 10.1128/mSphere.00090-17

**Published:** 2017-05-03

**Authors:** Isabelle Dietrich, Stephanie Jansen, Gamou Fall, Stephan Lorenzen, Martin Rudolf, Katrin Huber, Anna Heitmann, Sabine Schicht, El Hadji Ndiaye, Mick Watson, Ilaria Castelli, Benjamin Brennan, Richard M. Elliott, Mawlouth Diallo, Amadou A. Sall, Anna-Bella Failloux, Esther Schnettler, Alain Kohl, Stefanie C. Becker

**Affiliations:** a MRC-University of Glasgow Centre for Virus Research, Glasgow, Scotland, United Kingdom; b Bernhard-Nocht-Institut für Tropenmedizin, Hamburg, Germany; c Institut Pasteur de Dakar, Arbovirus and Viral Hemorrhagic Fever Unit, Dakar, Senegal; d German Mosquito Control Association (KABS/GFS), Waldsee, Germany; e Institut Pasteur de Dakar, Medical Entomology Unit, Dakar, Senegal; f The Roslin Institute, Royal (Dick) School of Veterinary Studies, Division of Genetics and Genomics, University of Edinburgh, Easter Bush, Edinburgh, United Kingdom; g Department of Virology, Arboviruses and Insect Vectors, Institut Pasteur, Paris, France; h Institute for Parasitology, University of Veterinary Medicine Hannover, Hannover, Germany; Boston University School of Medicine

**Keywords:** *Drosophila melanogaster*, RNA interference, Rift Valley fever virus, antiviral immunity, mosquito

## Abstract

The emerging bunyavirus Rift Valley fever virus (RVFV) is transmitted to humans and livestock by a large number of mosquito species. RNA interference (RNAi) has been characterized as an important innate immune defense mechanism used by mosquitoes to limit replication of positive-sense RNA flaviviruses and togaviruses; however, little is known about its role against negative-strand RNA viruses such as RVFV. We show that virus-specific small RNAs are produced in infected mosquito cells, in *Drosophila melanogaster* cells, and, most importantly, also in RVFV vector mosquitoes. By addressing the production of small RNAs in adult *Aedes* sp. and *Culex quinquefasciatus* mosquitoes, we showed the presence of virus-derived Piwi-interacting RNAs (piRNAs) not only in *Aedes* sp. but also in *C. quinquefasciatus* mosquitoes, indicating that antiviral RNA interference in *C. quinquefasciatus* mosquitoes is similar to the described activities of RNAi in *Aedes* sp. mosquitoes. We also show that these have antiviral activity, since silencing of RNAi pathway effectors enhances viral replication. Moreover, our data suggest that RVFV does not encode a suppressor of RNAi. These findings point toward a significant role of RNAi in the control of RVFV in mosquitoes.

**IMPORTANCE** Rift Valley fever virus (RVFV; *Phlebovirus*, *Bunyaviridae*) is an emerging zoonotic mosquito-borne pathogen of high relevance for human and animal health. Successful strategies of intervention in RVFV transmission by its mosquito vectors and the prevention of human and veterinary disease rely on a better understanding of the mechanisms that govern RVFV-vector interactions. Despite its medical importance, little is known about the factors that govern RVFV replication, dissemination, and transmission in the invertebrate host. Here we studied the role of the antiviral RNA interference immune pathways in the defense against RVFV in natural vector mosquitoes and mosquito cells and draw comparisons to the model insect *Drosophila melanogaster*. We found that RVFV infection induces both the exogenous small interfering RNA (siRNA) and piRNA pathways, which contribute to the control of viral replication in insects. Furthermore, we demonstrate the production of virus-derived piRNAs in *Culex quinquefasciatus* mosquitoes. Understanding these pathways and the targets within them offers the potential of the development of novel RVFV control measures in vector-based strategies.

## Abstract (precis)

Rift Valley fever virus (RVFV; *Phlebovirus*, *Bunyaviridae*) is an emerging zoonotic mosquito-borne pathogen of high relevance for human and animal health. Successful strategies of intervention in RVFV transmission by its mosquito vectors and the prevention of human and veterinary disease rely on a better understanding of the mechanisms that govern RVFV-vector interactions. Despite its medical importance, little is known about the factors that govern RVFV replication, dissemination, and transmission in the invertebrate host. Here we studied the role of the antiviral RNA interference immune pathways in the defense against RVFV in natural vector mosquitoes and mosquito cells and draw comparisons to the model insect *Drosophila melanogaster*. We found that RVFV infection induces both the exogenous small interfering RNA (siRNA) and piRNA pathways, which contribute to the control of viral replication in insects. Furthermore, we demonstrate the production of virus-derived piRNAs in *Culex quinquefasciatus* mosquitoes. Understanding these pathways and the targets within them offers the potential of the development of novel RVFV control measures in vector-based strategies.

## INTRODUCTION

Rift Valley fever (RVF; *Phlebovirus*, *Bunyaviridae*) is an important viral zoonosis in Africa. Rift Valley fever virus (RVFV) infection causes high fever, anorexia, and acute deaths in young ruminants and abortions in pregnant animals (1–3). In humans, symptoms are usually mild but infection can lead to retinitis, encephalitis, and hemorrhagic fevers. The case fatality rates have varied between epidemics but have averaged 1% overall (4–6).

RVFV was detected in at least 30 different mosquito species, of which several are known to transmit the virus (7), which complicates risk assessment of the spread of RVFV into new areas. Due to both the variety of vectors and the paucity of genetic tools for their analysis, the interaction between the vector and RVFV remains poorly characterized.

RVFV is encoded by a tripartite negative-sense or ambisense RNA genome. The large (L) segment encodes the RNA-dependent RNA polymerase. The medium (M) segment encodes the nonstructural protein NSm and envelope glycoproteins Gn and Gc as well as a 78-kDa protein and a 14-kDa protein which are expressed from alternative start codons (8). The small (S) segment encodes the nucleocapsid protein (N) and the nonstructural protein (NSs). These two proteins are expressed in an ambisense manner where the N protein is translated from an mRNA transcribed from the genomic RNA whereas the NSs protein is translated from an mRNA transcribed from the antigenomic S RNA (9, 10).

Competent vectors not only must allow virus infection and dissemination to saliva but also must control the adverse effects of virus replication. It is widely accepted that one key arthropod antiviral pathway is the exogenous small interfering RNA (exo-siRNA) pathway. Pioneering work in *Drosophila melanogaster* has demonstrated that this RNA interference (RNAi) pathway is initiated by the recognition and cleavage of double-stranded RNA (dsRNA) such as viral replication intermediates and possibly secondary RNA structures in the cytoplasm by the RNase III enzyme dicer-2 (Dcr2). This leads to production of 21-nucleotide (nt) virus-derived small interfering RNAs (viRNAs) and the sequence-specific degradation of viral genomes or transcription products by the multiprotein RNA-induced silencing complex (RISC), of which Argonaute-2 (Ago2) is the major component (11, 12). Orthologues of Dcr2 and Ago2 have also been identified in mosquitoes (13), and viRNAs have been shown to be produced in response to infection of mosquitoes with positive-strand RNA viruses of the *Togaviridae* and *Flaviviridae* families (14–16) but also for RVFV and other viruses in the *Bunyaviridae* family (17–19). Beyond induction of viRNAs, knockdown studies of RNAi proteins have shown that this pathway directly mediates antiviral activity (16, 20–23). Some viruses have evolved means to efficiently counteract the siRNA response. Viral suppressors of RNA silencing (VSRs) are diverse in sequence and mode of action and have been described for plant and insect viruses. For example, the NSs protein of the plant-infecting *Tospovirus* Tomato spotted wilt virus (TSWV; *Bunyaviridae*), the B2 protein of the insect flock house virus (FHV; *Nodaviridae*), and the VP3 protein of the mosquito-specific Culex Y virus (CYV; *Birnaviridae*) have been shown to sequester dsRNA molecules of different lengths, thereby inhibiting Dcr2-mediated cleavage of long dsRNAs into siRNAs and incorporation of small dsRNA species into RISC (19, 24–26). The dengue virus (DENV) NS4B protein was shown to have VSR activity. While it could not bind to dsRNAs, it interfered with dicing (27). A second arbovirus-targeting RNAi pathway is the Piwi-interacting RNA (piRNA) pathway. *D. melanogaster* and mosquito piRNAs are 24 to 29 nt in length and show a characteristic molecular signature (28–30). In *D. melanogaster*, piRNAs control the expression of transposons in germline cells and ovarian follicle cells and thereby help to sustain genomic integrity (28). They are generated in a Dicer-independent manner; instead, the piRNA pathway relies on Argonaute-3 (Ago3), Aubergine (Aub), and Piwi (28, 31, 32). Proposed mechanisms for piRNA production are the primary processing pathway and the so-called ping-pong amplification loop (33). Long single-stranded precursor RNAs are transcribed from piRNA clusters in the genome (28) and are processed to primary piRNAs via an unknown mechanism. Primary piRNAs are loaded into Aub and Piwi proteins to form so-called piRNA-induced silencing complexes (piRISCs) (34).

Interestingly, the Piwi protein clade has undergone an expansion in *Aedes* sp. and *Culex* sp. mosquitoes in contrast to *D. melanogaster*, which points to a diversification of piRNA pathway functions in mosquitoes besides transposon expression control. *A. aegypti* encodes seven Piwi proteins (Piwi1 to Piwi7) and Ago-3, which are sufficient to facilitate the production piRNAs and fulfill all functions of the pathway in *A. aegypti* despite the absence of Aub expression in these mosquitoes (13, 22). Arbovirus-specific piRNAs have been found in *Aedes* sp. mosquitoes and derived cells for a variety of arboviruses, including flaviviruses (DENV), alphaviruses (Sindbis virus [SINV], chikungunya virus, and Semliki Forest virus [SFV]), and bunyaviruses (29, 30, 35). However, how virus infection triggers piRNA production remains poorly understood. A recent study has given some insight into the mechanism of virus-derived synthesis of piRNAs in mosquito cells (36). Silencing of Piwi5 and Ago3 led to a significant reduction of secondary piRNAs in Sindbis virus (SINV, *Togaviridae*)- and DENV (*Flaviviridae*)-infected mosquito cells, which points to a functional role of these proteins in the ping-pong amplification cycle (36, 37). While Piwi5 was primarily associated with viral negative-strand-derived piRNAs with a U_1_ bias, Ago3 preferentially bound viral positive-strand-derived piRNAs, most of which displayed an A_10_ bias. It is therefore likely that Piwi5 and Ago3 act in tandem to generate virus-derived piRNAs. Further, silencing of Piwi4 in Semliki Forest virus (SFV, *Togaviridae*)- and Bunyamwera virus (BUNV, *Bunyaviridae*)-infected mosquito cells was shown to lead to increased viral replication, indicating that the piRNA pathway mediates antiviral activity (18, 22). The presence of RVFV-specific viRNAs and piRNAs in RVFV-infected mosquito cells and of viRNAs in *D. melanogaster* cells (17, 38) suggests an involvement of RNAi pathways also in the control of RVFV replication; however, their antiviral activity has not been confirmed.

In this study, we analyzed the induction and contribution of small-RNA pathways in controlling RVFV infection in multiple insect systems. Our results show that RVFV-specific small RNAs are produced in response to infection in several mosquito vector species, mosquito-derived cells, and *D. melanogaster* cells. We confirm that these act antivirally by silencing key proteins of the exogenous siRNA pathway in both *A. aegypti* Aag2 and *D. melanogaster* S2 cells and the piRNA pathway in Aag2 cells and through the use of luciferase-based, small-RNA-specific sensor constructs. Further to this, we found no experimental evidence for the presence of an RVFV suppressor of RNA silencing in the Aag2 and S2 cell systems.

## RESULTS

### Small-RNA production in RVFV-infected mosquito cells and *D. melanogaster* cells.

**FIG 1:**
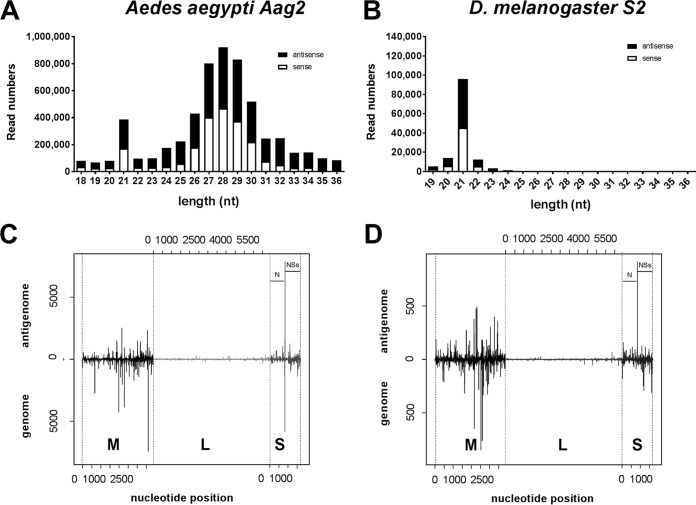
RVFV MP12-infected Aag2 and S2 cells produce virus-specific small RNAs. (A and B) Small-RNA reads matching the RVFV MP12 genomic/antigenomic sequences were plotted against the corresponding read lengths (in nucleotides [nt]) for (A) Aag2 cells infected for 24 h and (B) *D. melanogaster* S2 cells infected for 96 h prior to small-RNA extraction. (C and D) Alignment of the 21-nt small-RNA reads to the RVFV MP12 sequences of the three RNA segments. The *x* axis represents the position of small-RNA reads in reference to the antigenome RNA sequence (5′-to-3′ orientation) or the genome orientation (3′-to-5′ orientation). The *y* axis represents the number of reads for each position, with reads matching the antigenome shown on the positive *y* axis and reads matching the genome shown on the negative *y* axis. (C) Aag2 cells infected for 24 h. **(**D**)** *D. melanogaster* S2 cells infected for 96 h prior to small-RNA extraction.

To perform a comparative analysis of small-RNA pathways for RVFV infection in different insects, we set out to confirm that two key markers of an antiviral RNAi response occurred: (i) production of viral small RNAs and (ii) increased viral replication following knockdown of small-RNA pathway mediator proteins in *A. aegypti* and *D. melanogaster* cell lines. We infected *A. aegypti*-derived Aag2 cells and *D. melanogaster* S2 cells with RVFV strain MP12 and then carried out Illumina next-generation sequencing and analyzed the small-RNA fractions (Aag2 cells 24 h postinfection [p.i.], Fig. 1A; S2 cells 96 h p.i., Fig. 1B). We observed an enrichment of reads matching the RVFV MP12 genomic and antigenomic sequences at 21 to 22 nucleotides (nt) in Aag2 and S2 cells, whereas the enrichment of 27-nt to 30-nt sequences occurred exclusively in Aag2 cells. The 21-nt to 22-nt fraction most likely corresponds to viral siRNAs or microRNAs (miRNAs), whereas the 27-nt to 30-nt fraction reflects viral piRNAs. ViRNAs in Aag2 and S2 cells were derived from all three genome segments, with most viRNA reads per nucleotide matching the M segment sequence followed by the S segment and the L segment (Fig. 1C and D; see also Table S1 in the supplemental material) and mapped against viral genome and antigenome in equal ratios (Aag2, 0.98:1; S2, 1.2:1). Overall viRNA profiles in fly and mosquito cells were highly similar, pointing to conserved viRNA targeting and production in these different insect systems.

10.1128/mSphere.00090-17.3 TABLE S1  Analysis of viRNA reads from mosquito cells, adult mosquitoes, and *D. melanogaster*. The 21-nt viRNA reads for each sample were aligned to the three RVFV MP12 RNA segments, and data are given separately for the L, M, and S segments. Read numbers are given as total reads or reads relative to the length of the segment (reads per nt). Overrepresented segments of each sample are highlighted by a frame. The frequency of RVFV-derived viRNA reads (column 5) was calculated in reference to total read numbers. Download TABLE S1, DOCX file, 0.01 MB. Copyright © 2017 Dietrich et al. 2017 Dietrich et al. This content is distributed under the terms of the Creative Commons Attribution 4.0 International license.

**FIG 2:**
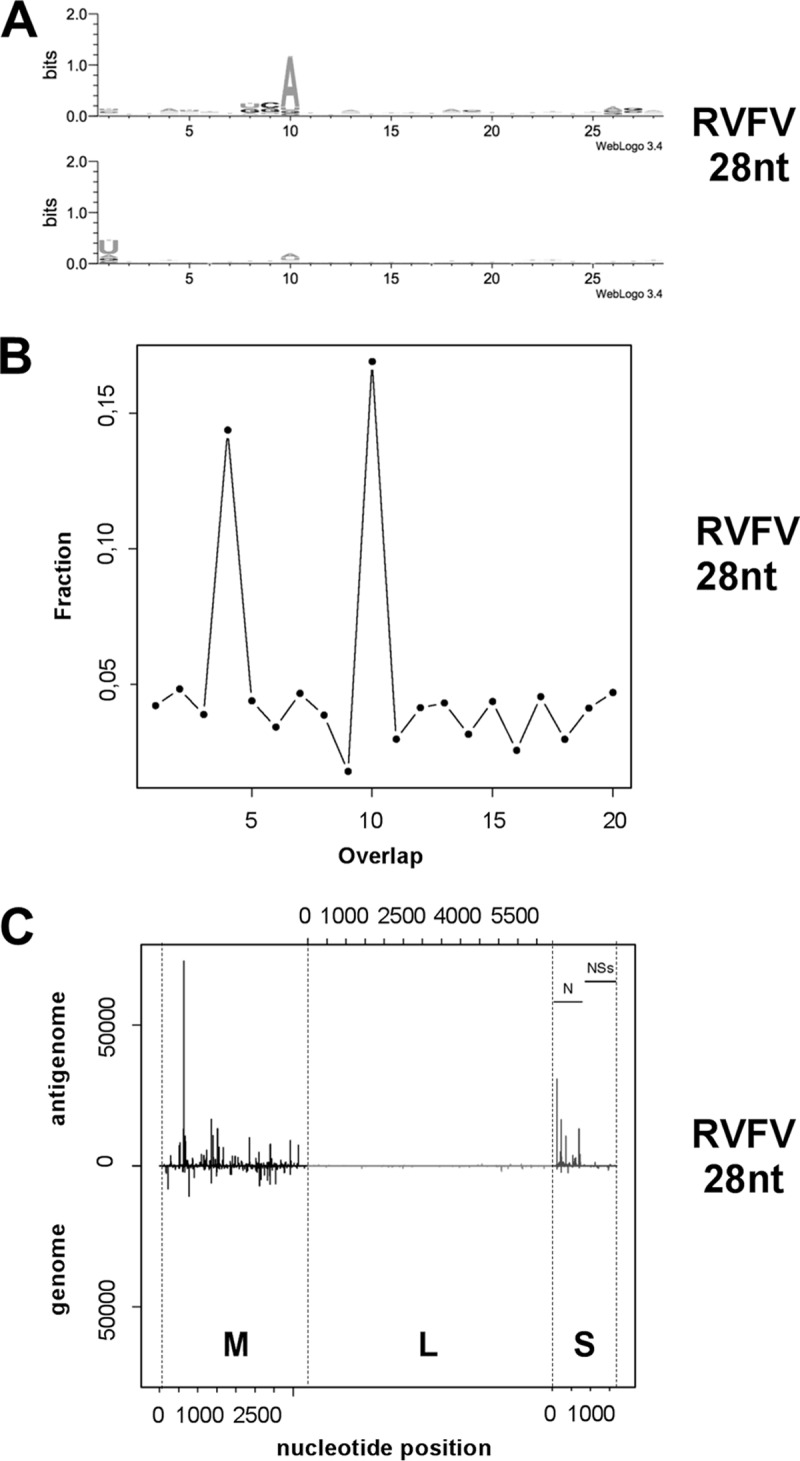
RVFV-specific piRNA-like small RNAs are produced in infected *Aedes aegypti* Aag2 cells. (A) Representation of piRNA sequence logos within the 28-nt reads. All reads matching the RVFV MP12 genomic and antigenomic sequences were analyzed for their base frequencies at each position. The *x* axis shows the nucleotide position. The *y* axis represents the frequency of each nucleotide at the corresponding position. (B) The panel shows the probability of overlap at the 5′ end of the opposite viral small RNA for the 28-nt reads. The *x* axis indicates the number of nucleotides overlapping between the antigenomic and genomic sequences; the *y* axis shows the mean fractions of reads overlapping. (C) Alignment of the 28-nt small-RNA reads to the RVFV MP12 sequences of the three viral RNA segments. The *x* axis represents the position of small-RNA reads in reference to the antigenome RNA sequence (5′-to-3′ orientation) or to the genome (3′-to-5′ orientation). The *y* axis represents the number of reads for each position, with reads matching the antigenome shown on the positive *y* axis and reads matching the genome shown on the negative *y* axis.

Next, we analyzed whether a piRNA signature was present within the 28-nt small-RNA reads mapping to the RVFV segments in Aag2 cells. A clear piRNA signature was found in sequence logos (A_10_ bias for antigenomic small RNAs and U_1_ bias for genomic small RNAs) and a 10-nt overlap of 5′-end genomic and antigenomic small RNAs as expected for piRNAs produced by the ping-pong amplification cycle (Fig. 2A and B). The 28-nt reads were also mapped against all three segments (Fig. 2C). Similarly to 21-nt viRNA results, coverage showed the read gradient M > S > L (Table S2). The ratio of genomic reads to antigenomic reads, however, showed an uneven distribution for the three segments (Table S2). For the M and S segments, we observed a clear enrichment of 28-nt piRNA-like molecules mapping to the antigenomic sequence. In contrast, a clear enrichment of genomic sequences was found within the L segment (Table S2).

10.1128/mSphere.00090-17.4 TABLE S2  Analysis of 28-nt piRNA reads from mosquito cells, adult mosquitoes, and *D. melanogaster*. The 28-nt piRNA reads for each sample were aligned to the three RVFV MP12 RNA segments, and data are given separately for the genomic and antigenomic sequences of the L, M, and S segments. The ratio shown in column 5 was calculated from these read numbers for the genomic and antigenomic sequences. Total read numbers and reads relative to length of the segment (reads per nt) are shown in columns 6 and 7. Overrepresented segments of each sample are highlighted by a frame. The frequency of RVFV-derived piRNA reads (column 8) was calculated in reference to total read numbers. Download TABLE S2, DOCX file, 0.02 MB. Copyright © 2017 Dietrich et al. 2017 Dietrich et al. This content is distributed under the terms of the Creative Commons Attribution 4.0 International license.

### Virus-derived small-RNA profiles in *Aedes* sp. and *Culex quinquefasciatus* mosquitoes.

**FIG 3:**
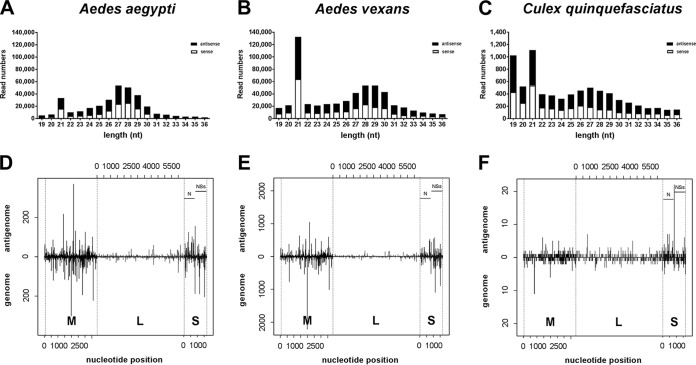
RVFV-derived small RNAs are present in three different vector mosquito species. The small-RNA fraction from RVFV MP12-infected *A. aegypti*, *A. vexans*, and *C. quinquefasciatus* whole mosquito bodies was isolated 14 days p.i. (*A. aegypti* and *C. quinquefasciatus*) or 15 days p.i. (*A. vexans*) and sequenced (A to C). The graphs represent the small-RNA reads mapping to the RVFV MP12 genomic and antigenomic sequences. Read numbers per length were plotted for *A. aegypti* (A), *A. vexans* (B), and *C. quinquefasciatus* (C) against the corresponding read length (in nucleotides) on a linear scale. (D to F) Alignment of the 21-nt small-RNA reads mapping to the RVFV MP12 sequences of the three RNA segments for all mosquito species as described for RVFV-derived small-RNA reads in Aag2 cells (Fig. 1C). The alignments for *A. aegypti* (D), *A. vexans* (E), and *C. quinquefasciatus* (F) are shown.

To verify virus-derived small-RNA production and patterns in RVFV vector mosquitoes, we infected three different mosquito species (*A. aegypti*, *A. vexans*, and *C. quinquefasciatus*) which have been associated with RVFV circulation in nature (39) and analyzed small-RNA profiles. RVFV-specific viRNAs (21 nt) and piRNA-like small RNAs (24 to 29 nt) were present in infected *A. aegypti* mosquitoes (Fig. 3A) and *A. vexans* mosquitoes (Fig. 3B). In both *Aedes* mosquito species, representation of RVFV-specific viRNAs reads was equal to or slightly greater than that seen with piRNA-like small RNAs. Both RVFV-specific viRNA and piRNA-like small-RNA read numbers were very low in infected *C. quinquefasciatus* mosquitoes (Fig. 3C) compared to both *Aedes* species. Similarly to Aag2 cell results, viRNA distribution in either *Aedes* species showed an enrichment of reads for the M segment and S segment and low read numbers for the L segment (Table S1). The relatively low RVFV-specific small-RNA read numbers in *C. quinquefasciatus* did not allow the establishment of a clear distribution pattern. ViRNAs from all three mosquito species were distributed along the three segments and mapped against the genome and antigenome (Fig. 3D to F).

**FIG 4:**
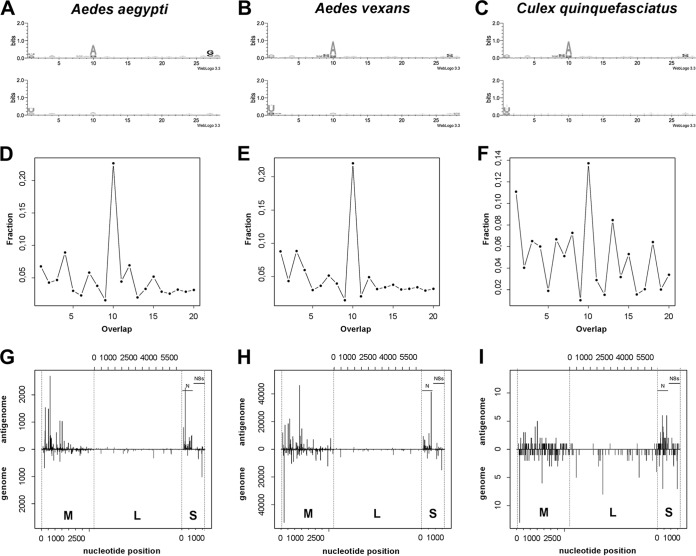
RVFV-specific piRNA-like small RNAs are produced in infected mosquitoes. (A to C) Representation of piRNA sequence logos within 28-nt reads. All reads mapping to the RVFV MP12 genomic and antigenomic sequences were analyzed for their base frequencies at each position for RVFV-derived small-RNA reads as previously described in Aag2 cells (Fig. 2A). *A. aegypti* data are presented in panel A, *A. vexans* data are shown in panel B, and *C. quinquefasciatus* data are shown in panel C. (D to F) The panels show the probability of overlap at the 5′ end of the opposite viral small RNA for the 28-nt reads. The *x* axis indicates the number of nucleotides overlapping between the antigenomic and genomic sequences; the *y* axis shows the mean fractions of reads overlapping. *A. aegypti* data are presented in panel D, *A. vexans* data are shown in panel E, and *C. quinquefasciatus* data are shown in panel F. (G to I) Alignment of the 28-nt small-RNA reads to RVFV MP12 RNA segments as previously described for RVFV-derived small-RNA reads in Aag2 cells (Fig. 2C). The alignment of *A. aegypti* is presented in panel G, that of *A. vexans* is shown in panel H, and that of *C. quinquefasciatus* is shown in in panel I.

**FIG 5:**
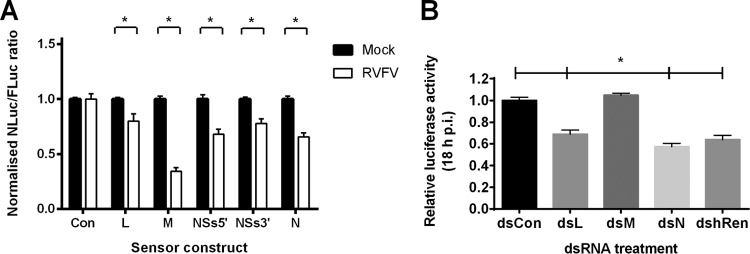
RVFV-derived small RNAs display antiviral properties. (A and B) The accessibility of RVFV mRNAs to RVFV-specific small RNAs and their antiviral properties were investigated using luciferase reporter-based sensor constructs (A) and RVFV-targeting dsRNAs (B), respectively. (A) Aag2 cells were either infected with RVFV MP12 (white bars) or left uninfected (black bars). At 24 h p.i., cells were transfected with small-RNA sensors (pIZ containing a nanoluciferase [NLuc] ORF fused to RVFV-specific/control sequences) and a transfection control (pIZ-FLuc). RVFV-specific sequences consisted of fragments amplified from the L open-reading frame (ORF; 515 nt), the M ORF (529 nt), the N ORF (498 nt), or the 5′ or 3′ halves of the NSs ORF (NSs5′, 416 nt; NSs3′, 329 nt). Primer binding sites for the respective fragments are indicated in Table S3. NLuc/FLuc ratios in transfected infected cells were normalized against the respective transfected uninfected cells. The graph presents means and standard errors of results from three independent experiments, each performed in triplicate. Statistical analysis was performed using the *t* test. *, *P* < 0.05. (B) dsRNAs targeting the RVFV rMP12delNSs:h*Ren* L, M, and S segments (dsL, dsM, dsN, dshRen) or a control dsRNA targeting eGFP (dsCon) were transfected into Aag2 cells, followed by virus infection. Viral replication was measured by luciferase assay, and dsL, dsM, dsN, and dshRen transfected samples were normalized against the control samples. The graph presents means and standard errors of results from three independent experiments, each performed in triplicate. Statistical analysis was performed using the *t* test. *, *P* < 0.05.

As for Aag2 cells, a piRNA signature was found in sequence logos of fragments for all three mosquito species (Fig. 4A to C) and 5′ end overlap of genome and antigenome 28-nt small RNAs (Fig. 4D to F). We also mapped the 28-nt reads from mosquitoes against the three genomic segments (Fig. 4G to I). Again, we found low coverage of the L segment; however, levels of coverage of the M and S segments were similar in all three mosquito species (Table S2). In *A. aegypti* mosquitoes, 28-nt piRNA-like molecules showed an enrichment of virus-derived sequences for the antigenomic sequence of the M segment. The same enrichment of antigenomic M segment sequences was not observed in *A. vexans* or *C. quinquefasciatus* (Table S2). In contrast, targeting of the antigenomic S segment sequence was a species-independent pattern present in all three mosquito species as well as Aag2 cells.

### Antiviral activity of RVFV-specific small RNAs.

The presence of RVFV-specific small RNAs in infected insect cells alone was not indicative of their antiviral activity, as they might have failed to become incorporated into RISC and might have been unable to actively target the viral RNA. Therefore, we wanted to confirm the functionality of virus-derived small RNAs produced during RVFV infection. To do so, small-RNA sensor constructs consisting of an OpiE2 insect promoter-driven luciferase reporter (nanoluciferase, NLuc) coding sequence fused to different 3′ untranslated, noncoding sequences originating from RVFV L and M segment mRNAs as well as N and NSs protein open-reading frames (ORFs) were used. If RVFV-specific, functional small RNAs were present in Aag2 cells following infection with RVFV, NLuc mRNA in the RVFV-specific small-RNA sensors would be degraded and NLuc expression reduced. Indeed, a reduction in luciferase activity was observed in Aag2 cells preinfected with RVFV (Fig. 5A), whereas luciferase expression from a control sensor containing an enhanced green fluorescent protein (eGFP) fragment in place of RVFV sequence was unaffected. In reverse, the ability of the exogenous siRNA machinery to target RVFV RNA during infection was tested using a recombinant RVFV that expresses humanized *Renilla* luciferase (h*Ren*) in place of the NSs gene (rMP12delNSs:h*Ren*; see the supplemental material). This reporter virus replicates efficiently in Aag2 cells although its replication is slightly impaired compared to that of the parental MP12 (Fig. S1A). Correlation of h*Ren* activity with viral replication was confirmed by comparison of luciferase enzyme activity (expressed as light units) to h*Ren* mRNA and viral N mRNA levels (Fig. S1B). Aag2 cells were transfected with dsRNAs against the RVFV L, M, and S segments prior to infection with rMP12delNSs:h*Ren*, and luciferase activity was measured 18 h p.i. (Fig. 5B); targeting of dsRNAs to the RNA-dependent RNA polymerase and the N and the h*Ren* mRNAs, but not the M mRNA, led to a significant reduction in RVFV replication. The failure of dsRNA targeting the M segment to reduce RVFV replication might reflect the fact that M segment gene products G_C_ and G_n_ do not directly participate in viral RNA replication and that viral replication can persist even under conditions in which glycoprotein expression is diminished.

10.1128/mSphere.00090-17.1 FIG S1  Validation of RVFVdelNSs:h*Ren* replication and gene knockdowns. The growth kinetics of rMP12delNSs:h*Ren* were characterized in Aag2 (A) and S2 (C) cells. Cells were infected at an MOI of 1, and virus growth was monitored at the indicated time points by plaque assay (A and C) or by luciferase activity and qRT-PCR (B). Aag2 cells (A) or S2 cells (C) were infected with MP12delNSs:h*Ren*, and cell supernatant was harvested at the indicated time points p.i. Plaque assays were performed, and the growth of rMP12delNSs:h*Ren* (black squares) was compared to that of RVFV MP12 (open circles). (B) To study the correlation between h*Ren* activity and viral replication, h*Ren* enzyme activity (expressed as light units; black circles) was compared to h*Ren* mRNA levels (open triangles) and viral N mRNA levels (black squares) at the indicated time points. (D) Impact of Ago2 knockdown on mRNA expression of RVFV MP12 in Aag2 cells. Knockdown of Ago2 was induced by transfection of dsRNA targeting the corresponding sequence (*A. aegypti* dsAgo2). Cells transfected with dsRNA targeting eGFP (dsCon) were used as controls. Cells were infected with MP12 at an MOI of 1 at 24 h p.t. Viral N mRNA levels were measured 24 h p.i. and normalized to *A. aegypti* ribosomal S7 RNA levels. The panel presents means and standard errors of results from three independent experiments, each performed in triplicate. Statistical analysis was performed using the *t* test. *, *P* < 0.05. (E) Knockdown of Ago2 in Aag2 cells was verified by qRT-PCR. The mRNA expression of Ago2 was normalized to S7 mRNA levels. Ago2 expression in dsAgo2-treated cells was normalized to that in control (dsCon-treated) cells. The panel presents means and standard errors of results from three independent experiments, each performed in triplicate. Statistical analysis was performed using the *t* test. *, *P* < 0.05. (F) The knockdown efficiency of Ago2 in S2 cells was verified by immunoblotting. Protein expression of Ago2 in cells transfected with Ago2-specific or control dsRNA was analyzed via Ago2 detection. Actin was used as a loading control. (G) Knockdowns of Piwi4, Piwi5, Piwi6, and Ago3 were verified by qRT-PCR. The mRNA expression levels of Piwi4, Piwi5, Piwi6, and Ago3 were normalized to *A. aegypti* ribosomal S7 RNA levels. Piwi4, Piwi5, Piwi6, and Ago3 expression in dsPiwi4-, dsPiwi5-, dsPiwi6-, and dsAgo3-treated cells, respectively, was normalized to that in control (dsCon-treated) cells. The panel presents means and standard errors of results from three independent experiments, each performed in triplicate. Statistical analysis was performed using the *t* test. *, *P* < 0.05. Download FIG S1, TIF file, 0.7 MB. Copyright © 2017 Dietrich et al. 2017 Dietrich et al. This content is distributed under the terms of the Creative Commons Attribution 4.0 International license.

In summary, here we have shown that RVFV-derived small RNAs from Aag2 cells were able to target homologous RNA fragments and are incorporated into the RISC, thus demonstrating their functionality.

### Antiviral effects of RNA interference in RVFV-infected Aag2 and S2 cells.

**FIG 6:**
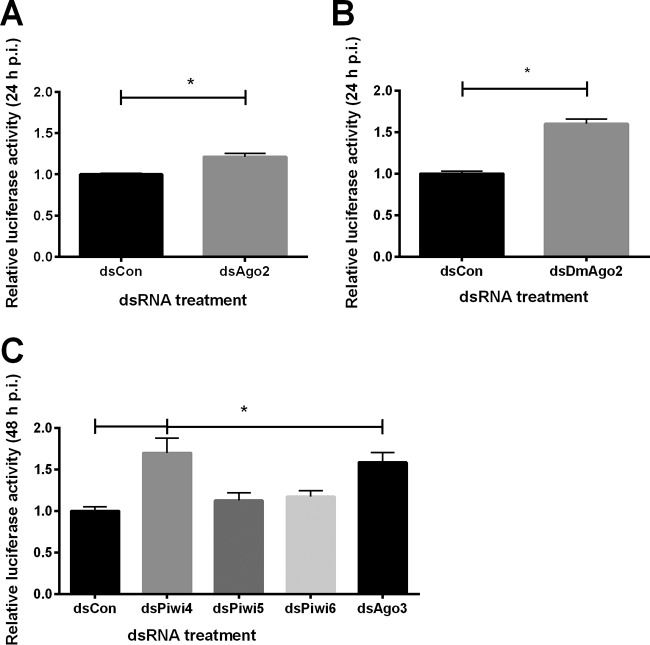
RNAi pathways limit replication of RVFV in *A. aegypti* and *D. melanogaster* cells. (A and B) Impact of Ago2 knockdown on replication of RVFV rMP12delNSs:h*Ren* in Aag2 cells (A) or* D. melanogaster* S2 cells (B). Knockdown of Ago2 was induced by transfection of dsRNA targeting the corresponding sequence (*A. aegypti* dsAgo2 in panel A or *D. melanogaster* dsDmAgo2 in panel B). Cells transfected with dsRNA targeting eGFP (dsCon) were used as controls. Viral replication was measured in dsAgo2-treated Aag2 and S2 cells by luciferase assay; values were normalized against the respective dsCon-treated cells. (A) The graph presents means and standard errors of results from five independent experiments performed in triplicate. (B) The graph presents means and standard errors of results from four independent experiments, each performed in sextuplicate. Statistical analysis was performed using the *t* test. *, *P* < 0.05. (C) Impact of Piwi4, Piwi5, Piwi6, and Ago3 knockdown on replication of RVFV rMP12delNSs:h*Ren* in Aag2 cells. Knockdown of Piwi4, Piwi5, Piwi6, and Ago3 was induced by transfection of dsRNA targeting the corresponding sequence, and dsRNA targeting eGFP (dsCon) was used as a control. Viral replication in dsPiwi4-, dsPiwi5-, dsPiwi6-, and dsAgo3-treated cells as measured by luciferase assay was normalized against that in control (dsCon-treated) cells. The panel represents mean values and standard errors of results from five independent experiments performed in triplicate. Statistical analysis was performed using the *t* test. *, *P* < 0.05.

Having demonstrated the presence and functionality of RVFV-derived small RNAs, we next analyzed the impact of silencing Ago2, a key component of the siRNA pathway, on RVFV replication in Aag2 and S2 cells and adult *D. melanogaster*. Aag2 and S2 cells were infected with the rMP12delNSs:h*Ren* virus strain, as described above. Similarly to Aag2 cells, rMP12delNSs:h*Ren* showed slightly impaired growth compared to rMP12 in S2 cells (Fig. S1C). Significantly enhanced viral replication, indicated by increased luciferase activity, was observed in Ago2 knockdowns in Aag2 and S2 cells (Fig. 6A and B). In addition to the data generated with reporter virus, we confirmed that RVFV MP12 replication was also enhanced following Ago2 knockdown (Fig. S1D). Silencing of Ago2 expression was verified by quantitative reverse transcription-PCR (qRT-PCR) in Aag2 cells (Fig. S1E) and Western blotting in S2 cells (Fig. S1F). With the fly model system established, we further analyzed the impact of Ago2 on RVFV infection *in vivo*. Wild-type (WT) flies (yw) and Ago2 null mutants were injected with RVFV MP12, and replication and survival of flies were measured. A significant increase in the levels of viral particles at day 5 after infection was observed in *Ago2*^*−/−*^ flies (Fig. S2A). Furthermore, the increased viral replication led to reduced survival of RVFV-infected *Ago2*^*−/−*^ mutant flies compared to mock-infected controls or RVFV-infected WT flies (Fig. S2B). Overall, these findings confirm a general role for the exogenous siRNA in insect antiviral responses to RVFV.

10.1128/mSphere.00090-17.2 FIG S2  Exogenous siRNAs limit RVFV infection and control survival in *D. melanogaster*. (A) Virus levels in samples at 5 days p.i. from wild-type (white) and *Ago2*^*−/−*^ (black) flies were verified by plaque assay. A log scale is used on the *y* axis for representation of virus titers; means and standard deviations from five independent experiments are presented. Statistical analysis of virus replication was performed using the Mann-Whitney test. *, *P* < 0.05. (B) Survival of wild-type and RNAi mutant flies after injection with RVFV MP12. The graph presents means and standard errors of results from five independent experiments (*n* = 20 per experiment) comparing wild-type (*yw*) and *Ago2*^*−/−*^ mutant *D. melanogaster* flies. Statistical analysis was performed using GraphPad Prism and the Mantel-Cox test. **, *P* < 0.01; ***, *P* < 0.0001. Download FIG S2, TIF file, 0.3 MB. Copyright © 2017 Dietrich et al. 2017 Dietrich et al. This content is distributed under the terms of the Creative Commons Attribution 4.0 International license.

Since we observed significant amounts of piRNAs in *A. aegypti* cells and whole mosquitoes, we further analyzed the impact of the piRNA pathway on antiviral defense by silencing of the pathway member proteins Piwi4, Piwi5, Piwi6, and Ago3 in Aag2 cells. Knockdown of Piwi4 and Ago3 led to an increase in viral replication, whereas knockdown of Piwi5 and Piwi6 did not significantly alter virus replication (Fig. 6C). Silencing was confirmed by qRT-PCR (Fig. S1G). These data suggest that the piRNA pathway plays a role in anti-RVFV defense in *A. aegypti*-derived cells.

### Does RVFV suppress RNAi?

**FIG 7:**
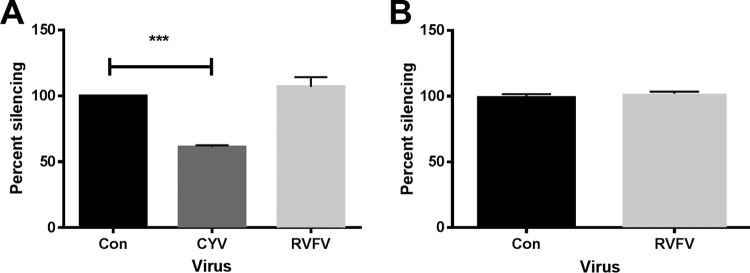
RVFV does not interfere with the exogenous RNAi pathway in mosquito and *D. melanogaster* cells. (A and B) The effect of RVFV MP12 protein expression on the induction of exogenous RNAi was analyzed using a FLuc reporter system. (A) Aag2 cells were infected with RVFV MP12 or Culex Y virus (CYV) as a control or left uninfected and subsequently transfected with luciferase expression plasmids (pIZ-FLuc as the reporter and pAct-Renilla as an internal control) and dsRNA targeting either FLuc or eGFP control sequences. Values of specific luciferase activity reduction were first normalized against a transfection control, and additional specific FLuc dsRNA-transfected samples were normalized against dseGFP-transfected controls. Effects of virus infection on RNAi induction are presented as percent silencing compared to the noninfected cells. The graph presents means and standard errors of results from three independent experiments performed in triplicate. Statistical analysis was performed using the *t* test. *, *P* < 0.05. (B) S2 cells were transfected with luciferase expression plasmids (pMT-Fluc as the reporter and pMT-Rluc as an internal control) and dsRNA targeting either FLuc or eGFP as a control. Cells were then either infected with RVFV MP12 or left uninfected. dsRNA targeting either FLuc or eGFP as a control was added to the cell culture media. The expression of luciferase reporters was induced and quantified. Data were analyzed and presented as described for panel A. The graph presents means and standard errors of results from three independent experiments performed in sextuplicate. Statistical analysis was performed using the *t* test. *, *P* < 0.05.

To determine whether RVFV encodes a suppressor of RNAi, we assessed the ability of virus to prevent dsRNA-mediated knockdown of a target mRNA (*Firefly* luciferase [FLuc]) in Aag2 (Fig. 7A) and S2 (Fig. 7B) cells. In both our assay systems, RVFV MP12 was unable to prevent dsRNA-based knockdown of the target reporter mRNA. This was in contrast to Culex Y virus (CYV, *Birnaviridae*), previously shown to encode a VSR protein (25), which inhibited FLuc silencing in Aag2 cells (Fig. 7A).

## DISCUSSION

Previous findings have demonstrated that viral RNA is a target for exogenous siRNA and piRNA pathways during RVFV ZH548 infection of mosquito cells (17). However, neither the functional relevance of these small RNAs nor the transferability of the result obtained in cells to whole mosquitoes has been demonstrated. In this study, we have expanded our understanding of RVFV-insect interactions by analyzing small-RNA profiles from RVFV MP12-infected *A. aegypti-*derived Aag2 cells and three different vector mosquito species (*A. aegypti*, *A. vexans*, and *C. quinquefasciatus*) as well as *D. melanogaster-*derived S2 cells. Furthermore, we tested the *in vivo* functionality of these small RNAs by the use of different cell-based reporter assays and the model insect *D. melanogaster*.

The viRNA profiles were derived from RVFV MP12-infected Aag2 cells, *Aedes* sp. mosquitoes, and *C. quinquefasciatus*. Mosquitoes and *D. melanogaster* S2 cells showed a large degree of similarity with regard to viRNA pattern and distribution but not with regard to viRNA abundance. We observed remarkably fewer viRNAs in *C. quinquefasciatus* mosquitoes than in *Aedes* sp. mosquitoes (see Table S1 in the supplemental material). This may either point to a species-related difference in levels of viRNA production efficiency (VPE) or be attributable to the infection status of individual mosquitoes used for small-RNA deep sequencing (for *A. vexans*, mean threshold cycle [*C*_*T*_] value of 21 and VPE level of 1.5% of total 21-nt fraction; for *A. aegypti*, mean *C*_*T*_ value of 26.6 and VPE level of 0.6%; for *C. quinquefasciatus*, mean *C*_*T*_ value of 27.8 and VPE level of 0.05%). Additional studies performed with viruses that target both *Aedes* sp. and *Culex* sp. mosquitoes are needed to clarify whether there are species-specific differences in levels of viRNA production. With regard to the viRNA pattern, the segment coverage levels and small-RNA ratios are highly similar between the different species tested and indicate that Dcr2-dependent dicing accounts for a considerable proportion of viRNAs in RVFV-infected insect cells and mosquitoes. These findings are in line with earlier small-RNA sequencing efforts in RVFV-infected Aag2 and S2 cells (17, 38) and in BUNV-infected Aag2 and *Culicoides sonorensis* KC cells (18) as well as with observations made in alphavirus- and flavivirus-infected insects (14, 16). Interestingly, we observed a favoring of the M segment over the S segment despite the fact that the S segment is the most abundant segment, at least in infected mammalian cells (L segment/M segment/S segment ratios = 1:3:13 [40]). With regard to segment favoring, our data also contrast with previous observations made in BUNV-infected Aag2 and KC cells, where the S segment is favored (18). The overrepresentation of M segment reads in RVFV-infected cells and *Aedes* sp. mosquitoes and *D. melanogaster* indicates a biological significance of this pattern; however, the data are difficult to interpret with current knowledge, since the ratios of L, M, and S segments in insect cells have not been analyzed. Furthermore, we observed the same bias toward the intergenic region (IGR) previously reported by Sabin et al. (38) in S2 cells and also in mosquito cells and adult mosquitoes. Interestingly, this bias was seen only for the antigenome IGR sequence in S2 cells (reference 38 and our study) and *C. quinquefasciatus*, whereas it was observed for both the genome and the antigenome in infected mosquito cells and *Aedes* sp. mosquitoes. We speculate that hybridization of complementary N and NSs mRNAs or their overlapping transcription termination signals (41–43) provides an easily accessible Dcr2 substrate. In contrast, the partially double-stranded genomic panhandle structures formed by the termini would require protection from Dcr2 degradation in insect cells and are thus covered by N protein. The apparent differences between species (fly and *C. quinquefasciatus* mosquitoes versus *Aedes* sp. mosquitoes) or virus strains (MP12 strain versus ZH548 [17]) may be due to tissue-specific effects present under *in vivo* conditions but could also be due to viruses in multiple phases of replication in live mosquitoes which could mask more-specific effects seen in the relatively synchronized infection of cell lines. The possibility that *in vivo* targeting and *in vitro* targeting function differently cannot be excluded at present, and future studies need to take this into account. Alternatively, the conflict may have been due to technical issues such as library preparation (44). Nonetheless, as sequencing was carried out independently in two locations (Hamburg and Glasgow), we argue that this targeting represents a real signature of viRNA production present during infection of insect cells with RVFV MP12.

It has previously been speculated on the basis of superinfection exclusion assays that small RNAs may mediate antiviral activity (17). Nonetheless, a direct demonstration of an antiviral role of small-RNA pathways against RVFV has been missing, and our data demonstrate a key role for the exogenous siRNA and piRNA pathways in controlling virus replication. We show that RVFV cannot be targeted by artificially induced siRNAs only and that infection produces virus-derived small RNAs with antiviral activity, that Ago2, Piwi4, and Ago3 are involved in the antiviral response, and that RVFV is unable to inhibit at least dsRNA-induced RNAi.

Given the availability of a number of well-characterized genetic *D. melanogaster* models which allow *in vivo* studies and facilitate high-throughput studies for host factor identification (45–47), we used the fruit fly to demonstrate that defects in the exogenous RNAi pathway (specifically in Ago2) lead to increased viral replication and thus to higher fly mortality. This suggests a link between processing of small RNAs derived from the RVFV RNA segments (17, 38) and antiviral defense in *D. melanogaster*.

In addition to the 21-nt viRNAs, virus-specific piRNA-like molecules of 24 to 30 nt in length were detected in all RVFV-infected mosquitoes and cells but not in *D. melanogaster* cells. The presence of arboviral piRNA-like molecules in mosquitoes is in line with previous reports of RVFV-infected mosquito cell lines (17), as well as other bunyaviruses and alphaviruses (18, 22, 29, 30, 35, 48), whereas their absence in *D. melanogaster* has been recently reported (49). Most characteristics of the RVFV-specific piRNA-like molecules are shared between all tested mosquitoes and the Aag2 cells, including the unbalanced production across the different segments (M > S > L) and the bias toward reads mapping to the M and S segment antigenomes. The preference for production of piRNA-like reads matching the antigenome of negative-strand viruses has been previously reported for La Crosse virus (LACV, *Bunyaviridae*) in C6/36 cells (for all three segments) and for RVFV ZH548 infection in different mosquito cells (only for the S segment) (17, 30, 48). The bias of piRNA-like molecules mapping to the antigenome of RVFV cannot be explained by the amount of antigenome versus genome during an infection, as recent research has shown that approximately 2.5-fold to 4.5-fold more genome RNA than antigenome RNA is present during RVFV MP12 infection of different cells (50). However, as stated above, it can be expected that viral mRNAs would be more accessible to cytoplasmic components of RNAi pathways than encapsidated genomic/antigenomic RNAs. It is difficult to distinguish between the viral mRNA and antigenome species in the case of the RVFV L and M segments in contrast to the ambisense S segment. As recently shown, more N mRNA than NSs mRNA is present during RVFV MP12 infection (50), which is in line with the bias of piRNAs mapping to the antigenome of the N gene observed in infected mosquitoes and Aag2 cells at different time points. However, a similar bias was not observed for RVFV ZH548 infection of Aag2 cells (17). Whether this was due to experimental setup or strain-specific issues is not known at this point. The production of these piRNA-like molecules during RVFV infection and the increase of RVFV replication in Aag2 cells upon knockdown of Piwi4 and Ago3 strongly support the idea of an antiviral function of the piRNA pathway in addition to the exogenous siRNA pathway during RVFV infection of mosquitoes. Similar results have been reported for the positive-strand alphaviruses (16, 22). Taking the data together, it can be hypothesized that the mosquito piRNA pathway can target positive-strand and negative-strand RNA arboviruses. The detection of RVFV-specific piRNA-like molecules in infected *C. quinquefasciatus* mosquitoes suggests that the antiviral function of the piRNA pathway is not restricted to *Aedes* sp. mosquitoes but can be extended to *Culex* sp. mosquitoes.

In summary, we show that antiviral RNAi pathways—the exogenous siRNA pathway and the piRNA pathway—are induced by RVFV infection across a number of vector species and the fly model and that RVFV is unable to prevent dsRNA-induced RNAi. Thus, this segmented negative-strand RNA virus is very similar to positive-stranded RNA arboviruses with respect to targeting by mosquito RNAi responses. The universal role of RNAi pathways points to their importance in combating virus infection throughout insects and makes them an attractive target for manipulation.

## MATERIALS AND METHODS

### Animal ethics statement.

The Institut Pasteur de Dakar (IPD) has received authorization from the Senegalese Ministry of Health to perform experiments on live animals in accordance with international regulations, and policies governing the care and use of laboratory animals and the IPD are guided by the *International Guiding Principles for Biomedical Research Involving Animals* developed by the Council for International Organizations of Medical Sciences.

### Tissue culture and virus production.

BHK-21 cells were grown in Leibovitz (L-15) media or Glasgow’s minimal essential medium (GMEM) supplemented with 10% calf serum, 100 U/ml penicillin, and 100 µg/ml streptomycin. Vero E6 cells were maintained in Leibovitz (L-15) media supplemented with 10% fetal calf serum, 100 U/ml penicillin, and 100 µg/ml streptomycin. Cells were grown at 37°C in 5% CO_2_. *A. aegypti*-derived Aag2 cells were grown in L-15 media supplemented with 10% fetal calf serum, 100 U/ml penicillin, 100 µg/ml streptomycin, and 10% tryptose phosphate broth at 28°C. *D. melanogaster* S2 cells were propagated in Schneider’s media supplemented with 10% fetal calf serum, 100 U/ml penicillin, and 100 µg/ml streptomycin at 28°C. Propagation of RVFV strain MP12 was performed on BHK-21 cells at 33°C, unless stated otherwise, and virus titers were measured by plaque assay on Vero E6 cells (BNI) or BHK-21 cells (CVR).

### Construction of an RVFV reporter virus and virus rescue.

In order to monitor RVFV replication in insect cells, a reporter virus expressing luciferase in place of the NSs ORF was constructed (51). To delete the NSs gene in the RVFV MP12 S segment, plasmid pTVT7-GS (52) was used as the template in an excision PCR (53), using outward-facing forward and reverse primers (see Table S3 in the supplemental material). The PCR product generated lacked nucleotides 19 to 819 of the RVFV MP12 S segment and possessed at its 3′ end SpeI and KpnI restriction sites and at its 5′ end KpnI and PmII sites. The PCR product was digested with KpnI and religated to form plasmid pTVT7-GSΔNSs-*Kpn*I. Digestion of pTVT7-GSΔNSs-*Kpn*I with PmlI and SpeI allowed directional insertion of humanized *Renilla* luciferase (h*Ren*) into the S segment in an ambisense orientation to generate pTVT7-GSΔNSs:h*Ren*. The coding sequence for h*Ren* was amplified by PCR from phRL-CMV (phRL-cytomegalovirus) (Promega). Rescue of the rMP12ΔNSs:h*Ren* reporter virus was performed using a reverse genetics system described earlier (54). Briefly, 7 × 10^5^ BSR-T7/5 cells were seeded in a T-25 flask and left to adhere overnight. Cells were transfected with expression plasmids pTM1-L (0.5 μg) and pTM1-N (0.5 μg) together with 1 μg each of transcription plasmids pTVT7-GL, pTVT7-GM, and pTVT7-GSΔNSs:h*Ren* using Lipofectamine 2000 (Life Technologies, Inc.). Virus-containing supernatant was harvested when extensive cytopathic effect was observed in infected-cell monolayers (typically 5 to 7 days p.i.), clarified by centrifugation, and stored at −80°C.

10.1128/mSphere.00090-17.5 TABLE S3  Oligonucleotides used for PCR, cloning, and qRT-PCRs. Sequences of all oligonucleotides are sorted by application. For all oligonucleotides not based on commercial vectors, the gene accession number and genome position are given. Download TABLE S3, DOC file, 0.1 MB. Copyright © 2017 Dietrich et al. 2017 Dietrich et al. This content is distributed under the terms of the Creative Commons Attribution 4.0 International license.

### Reporter virus characterization in Aag2 cells.

rMP12delNSs:h*Ren* growth curves were performed in Aag2 cells. For this, 1.7 × 10^5^ Aag2 cells per well were seeded in 24-well plates and left to adhere overnight. Cells were infected at a multiplicity of infection (MOI) of 1, and virus growth was monitored at the indicated time points by plaque assay, luciferase activity assay, and qRT-PCR. Plaque assays were performed using cell supernatant as described above. Infected cell monolayers were lysed using passive lysis buffer (Promega). Luciferase assays were performed using *Renilla*-Glo substrate (Promega) on a GloMax luminometer. For qRT-PCR, cells were lysed in TRIzol reagent (Life Technologies, Inc.). qRT-PCRs were performed to determine rMP12delNSs:h*Ren* h*Ren* and nucleocapsid (N) mRNA copy numbers in infected Aag2 cells using random hexamer primers and a QuantiTect Probe PCR kit according to the protocol of the manufacturer (Qiagen) with the following primers and probe: for hRen FP, hRen RP and hRen probe (h*Ren*); for RVF FP, RVF RP and RVF probe (RVFV N) (Table S3). *A. aegypti* ribosomal S7 was amplified as an internal control using primers Aag-S7 FP and Aag-S7 RP and probe Aag-S7-Probe (Table S3).

### Mosquito maintenance and infections.

*A. aegypti* (Linneaus) and *C. quinquefasciatus* (Say) laboratory strains (Bayer) were housed in environmental chambers with 25°C ± 2°C average temperature, 80% relative humidity, and 12-h photoperiodicity. *A. aegypti* eggs were dried for 2 weeks before they were floated in dechlorinated water. *C. quinquefasciatus* eggs were floated directly after deposition. Larvae were kept in dechlorinated tap water and were fed on TetraMin baby fish food (Tetra). Fourth-instar larvae were transferred into separate boxes and maintained in adult cages (Bioquip) (60 by 60 by 60 cm). A sugar solution (10% fructose) was used as the carbohydrate source to feed adult mosquitoes. Female mosquitoes were fed with human donor blood provided on a cotton patch, a method that has been demonstrated to give results similar to those of membrane feeding in *Culex* species and *Aedes* species mosquitoes (55). For infection experiments, 4-to-7-day-old females were separated into 175-ml plastic tubes (Greiner Bio-One) and starved 24 h prior to provision of an infectious blood meal. The infectious blood meal was offered on a cotton patch and contained 1 × 10^7^ PFU/ml RVFV MP12 mixed with human donor blood. Females were allowed to feed for 3 h at 28°C in the incubator. After feeding, fully engorged females were sorted and kept for 14 days at 28°C and 80% humidity. To confirm the positive infection status of mosquitoes, one leg was removed and tested by RVFV L segment-specific qRT-PCR as described below. Only females with *C*_*T*_ values below 30 were kept for further analysis. The remaining bodies of females with positive infection status (*A. aegypti*, *n* = 10; *C. quinquefasciatus*, *n* = 10) were pooled, and total RNA was isolated using TRIzol reagent (Life Technologies, Inc.) according to the manufacturer’s instructions.

*A. vexans* mosquitoes were collected from a ground pool in Barkédji village (15°17′N, 14°53′W), in the Ferlo region of Senegal. The F1 generation adult mosquitoes used for the experimental infection were reared in the laboratory under standard conditions at 27 ± 1°C and a relative humidity of 70% to 75% (56). RVFV MP12 strain viral stock was prepared and mosquito infections were performed as previously described (57). Female mosquitoes (5 to 7 days old) were starved for 48 h prior to exposure to an infectious blood meal for 1 h. The blood meal consisted of a 33% volume of washed rabbit erythrocytes, a 33% volume of viral stock (1 × 10^8^ PFU/ml), a 20% volume of fetal bovine serum (FBS), a 10% volume of sucrose, 5 mM ATP, and 150 to 250 mg of sodium bicarbonate. The blood meal was administered using chicken skins as membrane feeders. After feeding, a sample of remaining blood meal was stored at −80°C for titration. The mosquitoes were then cold anesthetized, and engorged mosquitoes were incubated at 27°C, with relative humidity of 70% to 80%, and fed with 10% sucrose. At 15 days postfeeding, mosquitoes were cold anesthetized. For each mosquito, legs and wings were removed and collected in single tubes; bodies were stored in separate tubes. Samples were kept at −80°C until their analysis. Leg and wing samples were screened by qRT-PCR for detection of virus using the following procedure. Each mosquito’s legs and wings were titrated in 500 µl of L-15 media supplemented with 10% heat-inactivated fetal bovine serum, using a homogenizer. The suspensions were clarified by centrifugation (5,000 × *g* for 10 min at 4°C), and the supernatant was used for RNA extraction with a QIAamp viral RNA minikit (Qiagen), according to the manufacturer’s protocols. The RNA samples were screened for RVFV presence by qRT-PCR using primers RVF FP and RVF RP and an RVF probe (Table S3). The qRT-PCR assay was performed using an ABI 7500 cycler (Applied Biosystems) and a QuantiTect Probe RT-PCR kit (Qiagen). A total of 11 bodies corresponding to infection-positive legs/wings with *C*_*T*_ values below 30 were pooled in TRIzol reagent for RNA extraction.

RVFV strain MP12 has been generated through serial passage on MRC-5 cells in the presence of 5-fluorouacil, which generated mutations in all segments leading to attenuation of the virus in mammalian hosts, which may in part be linked to temperature sensitivity (58) However, previous studies have demonstrated that there is no difference in the levels of virus replication and dissemination between MP12 and virulent variant ZH501 in *Culex pipiens* mosquitoes (59).

### Fly culture and infection.

*D. melanogaster* strains *yw* and *Ago2*^414^ have been previously described (60). The fly stocks were raised on standard cornmeal-agar medium (Carolina) at 20°C. Adult flies of 4 to 6 days of age were used for infection experiments. Infections were carried out by intrathoracic injection (Nanoject II apparatus; Drummond Scientific) of 10 nl of a viral suspension (RVFV at 5 × 10^8^ PFU/ml) in cell culture media. Injection of the same volume of sterile cell culture media was used as a control. Infected flies were then incubated at 25°C on Nutri-Fly Bloomington Formulation (Flystuff).

### RVF virus titration from infected flies.

Flies were collected at 5 days p.i., pooled (five specimens), placed in sterile 1.5-ml tubes, and triturated in 500 µl of cell culture medium (high-glucose Dulbecco’s modified Eagle’s medium [Sigma-Aldrich]) containing 10% heat-inactivated fetal bovine serum, 100 U/ml penicillin, and 100 µg/ml streptomycin, using a tissue homogenizer. The suspensions were clarified by centrifugation (5,000 × *g* for 1 min), and the supernatant was used for virus titration on Vero E6 cells.

### Small-RNA deep sequencing.

Small-RNA sequencing from Aag2 cells and mosquitoes was carried out by Edinburgh Genomics (University of Edinburgh) using an Illumina HighSeq 2000 platform and a HighSeq 2500 platform, respectively, as previously described (18). Cells were either infected with RVFV strain MP12 at a multiplicity of infection (MOI) of 5 or left uninfected. At 24 h p.i., RNA was isolated using 1 ml TRIzol reagent (Life Technologies, Inc.) per flask followed by purification.

Similarly, S2 cells were infected at an MOI of 1, and the small-RNA fraction was isolated 96 h p.i. using a mirVana miRNA isolation kit (Ambion). The small-RNA fraction derived from purification was further size selected by fractionation on a 15% PAGE gel (7 M urea, 0.5× Tris-borate-EDTA [TBE]). RNAs (18 to 24 nucleotides in size) were excised using a RiboReady Colour Micro RNA ladder (Amresco) for size control and eluted from gel overnight in 0.3 M NaCl buffer at 4°C with horizontal shaking (200 rpm). Purified 18-nt to 24-nt small-RNA fractions were suspended in 10 μl H_2_O, and library preparation was performed using an NEB Next Multiplex small RNA Library Prep Set for Illumina (New England Biolabs) according to the manufacturer’s protocol. Sequencing was performed using a MiSeq reagent kit (v3) and an Illumina MiSeq platform according to the manufacturer’s protocol.

Analysis of small-RNA reads was performed by the use of clipping adapters, and identical sequences were collapsed using the fastx toolkit (http://hannonlab.cshl.edu/fastx_toolkit/). Reads were then aligned to RVFV (NCBI accession numbers: for the L segment, DQ375404; for the M segment, DQ380208; for the S segment, DQ380154) using BWA-backtrack (62). Length distributions of the reads and positioning on the RVFV genome were analyzed using in-house perl (version 5.20.1) and R (63) (version 3.1.1) scripts. To examine the preferred overlap between reads mapping on opposite strands, for each position of the genomic strand, the numbers of reads on the antigenomic strand with overlaps of between 1 and 20 nucleotides were counted, normalized to 1, and summed up (weighted with the numbers of reads mapping at the respective positions of the genomic strand).

### *In vitro* dsRNA production.

dsRNA molecules targeting *A. aegypti* Dcr2, Ago2, Piwi4, Piwi5, Piwi6, and Ago3 and *D. melanogaster* Ago1 and Ago2 as well as eGFP were produced with a T7 RNA polymerase *in vitro* transcription kit (Megascript RNAi kit; Ambion) using PCR products as the template. *A. aegypti*, Ago2, Piwi4, Piwi5, Piwi6, and Ago3 PCR products were obtained from cDNA derived from Aag2 cell total RNA. *D. melanogaster* Ago2 PCR products were generated from S2 cell total RNA. eGFP was amplified from pEGFP-C1 (Clontech). Primers were used as described previously (18, 20, 22, 61).

### Silencing of RNAi components.

Aag2 cells were seeded at a density of 1.7 × 10^5^ cells/well in 24-well plates and left to adhere overnight. Cells were transfected with 300 ng of dsRNA targeting *A. aegypti* Ago2 (dsAgo2), Piwi4 (dsPiwi4), Piwi5 (dsPiwi5), Piwi6 (dsPiwi6), and Ago3 (dsAgo3) using Lipofectamine 2000 (Life Technologies, Inc.) according to manufacturer’s specifications. dsRNA against eGFP (dseGFP) was used as a control. At 24 h posttransfection (p.t.), cells were infected with recombinant RVFV rMP12delNSs:h*Ren* at an MOI of 0.1 (Ago2 knockdowns) or 0.01 (Piwi and Ago3 knockdowns). At 24 h p.i. (Ago2 knockdowns) or 48 h p.i. (Piwi and Ago3 knockdowns), luciferase activity was determined using *Renilla*-Glo substrate (Promega) on a GloMax luminometer following cell lysis in passive lysis buffer. Knockdown of Ago2, Piwi4, Piwi5, Piwi6, and Ago3 was confirmed by qRT-PCR as previously described (18, 20). *A. aegypti* ribosomal S7 was used as an internal control for relative quantifications.

Similarly, Ago2 silencing was performed in S2 cells. S2 cells were seeded at a density of 5 × 10^4^ in 96-well plates and left to adhere overnight. A 25-ng volume of dsRNA targeting *D. melanogaster* Ago2 (dsDmAgo2) (61) was then added to the cells. Infections and luciferase assays were performed as described for Aag2 cells. Ago2 knockdown was confirmed by immunoblotting using anti-Ago2/eIF2C2 (AB5072) antibodies (Abcam, Inc.). Actin was used as a loading control and detected using an anti-actin antibody (clone JLA20; Millipore).

### dsRNA-mediated silencing of RVFV.

dsRNA molecules targeting rMP12delNSs:h*Ren* or eGFP were produced as described above. PCR products were generated using plasmids pTVT7-GL, pTVT7-GM, and pTVT7-GS and primers dsL-Fwd and dsL-Rev, primers dsM-Fwd and dsM-Rev, and primers dsN-Fwd and dsN-Rev, respectively (Table S3). The h*Ren* dsRNA PCR template was amplified from pGL4.75 (Promega) using primers dshRen-Fwd and dshRen-Rev (Table S3). Transfections of Aag2 cells were performed as described above. At 4 h (p.t.), cells were infected with rMP12delNSs:h*Ren* at an MOI of 0.1. At 18 h p.i., luciferase activity was determined using *Renilla*-Glo substrate (Promega) on a GloMax luminometer following cell lysis in passive lysis buffer.

### Small-RNA sensor assays.

Small-RNA sensors were constructed by cloning nanoluciferase (NLuc) cDNA into the pIZ vector (Life Technologies, Inc.) using HindIII and XbaI restriction sites. NLuc was amplified from pNL1.1 vector (Promega) using primers Nanoluc-Fwd and Nanoluc-Rev (Table S3). RVFV MP12 fragments were amplified from plasmids pTVT7-GL, pTVT7-GM, and pTVT7-GS and inserted downstream of the NLuc ORF using XbaI and SacII restriction sites and the following primers: primers RVFV L F XbaI and RVFV L R SacII; primers RVFV M Fwd XbaI and RVFV M Rev. Sac II; primers RVFV N FwdXbaI and RVFV New Rev SacII; primers NSs5′ F XbaI and NSs5′ R SacII; and primers NSs3′ F XbaI and NSs3′ R SacII (Table S3). RVFV-specific fragment lengths were as follows: for L ORF, 515 nt; for M ORF, 529 nt; for N ORF, 498 nt; for NSs ORF 5′ half (NSs5′), 416 nt; for NSs ORF 3′ half (NSs3′), 329 nt. Primer binding sites for the respective fragments are indicated in Table S3. A fragment of the eGFP cDNA was amplified from pEGFP-C1 (Clontech) using primers eGFP-XbaI-FW and eGFP-SacII-RV (Table S3) and inserted into pIZ-NLuc using XbaI and SacII restriction sites. Aag2 cells (1.7 × 10^5^ per well) were seeded into 24-well plates and left to adhere overnight. Cells were either infected with RVFV MP12 at an MOI of 1 or left uninfected. At 24 h p.i., all cells were transfected with 200 ng of the different small-RNA sensors using Lipofectamine 2000 transfection reagent. pIZ (100 ng) expressing *Firefly* luciferase (pIZ-Fluc) (64, 65) was used as a transfection control. At 24 h p.i., NLuc and FLuc expression was measured using Nano-Glo and Bright-Glo substrates (Promega).

### RNAi suppression assays.

RNAi suppression assays were performed in both Aag2 and S2 cells. Aag2 cells (1.7 × 10^5^ per well in a 24-well plate) were infected with RVFV MP12 or the positive-control CYV at an MOI of 5 and incubated for 24 h at 28°C. Cells were then cotransfected with 100 ng pIZ-Fluc (64, 65), 40 ng pAct-Renilla (66) (FLuc and RLuc expression plasmids, respectively), and 0.05 ng dsRNA targeting FLuc or a control dsRNA targeting eGFP using Lipofectamine 2000. At 24 h p.t., cells were lysed in passive lysis buffer and FLuc and RLuc expression was determined using Bright-Glo and *Renilla*-Glo substrates (Promega).

RNAi suppression assays in S2 cells have previously been described (67, 68). Briefly, 5 × 10^4^ S2 cells were seeded in 96-well plates and left to adhere overnight. Cells were transfected with 50 ng pMT-Fluc and 15 ng pMT-Rluc for 24 h at 28°C, after which the cells were either infected with RVFV MP12 at an MOI of 5 for 24 h or left uninfected. A 25-ng volume of dsRNA targeting either FLuc or eGFP as a control was fed to the cells. At 7 h after the feeding of dsRNA, expression of luciferase reporters was induced by addition of 0.5 mM CuSO_4_ per well. After 24 h, cells were lysed and luciferase expression was determined as described above.

### Statistical analyses.

All statistical analyses presented were performed using GraphPad Prism (version 5; GraphPad Software, Inc., La Jolla, CA, USA). Survival analysis was carried out with the Kaplan-Meier test and the log rank test. Statistical analyses of the data from dsRNA knockdown experiments and small-RNA sensor and RNAi suppressor assays were performed using the *t* test. *P* value representations for all tests are as follows: *, *P* = <0.05; **, *P* = <0.01; ***, *P* = <0.001; ****, *P* = <0.0001.

### Accession number(s).

Small RNA sequencing data have been deposited in the NCBI database under accession no. PRJNA383480.
