# Supplementary material for: RNA Interference Restricts Rift Valley Fever Virus in Multiple Insect Systems
Source: mSphere. 2017 May 3;2(3):e00090-17. doi: 10.1128/mSphere.00090-17 (PMC5415632; doi:10.1128/mSphere.00090-17)
Supplement: TABLE S1 [file sph002172276st3.docx]

| **Sample** | **Segment** | **total reads** | **reads per nt** | **% RVFV 21nt** |
| --- | --- | --- | --- | --- |
| Aag2 cells | L | 38808 | 6.06 |  |
| Aag2 cells | M | 241888 | 62.25 |  |
| Aag2 cells | S | 52924 | 31.30 | 2.40 |
| *D. melanogaster* S2 | L | 5999 | 0.94 |  |
| *D. melanogaster* S2 | M | 69405 | 17.86 |  |
| *D. melanogaster* S2 | S | 9245 | 5.47 | 4.10 |
| *Aedes aegypti* | L | 5447 | 0.85 |  |
| *Aedes aegypti* | M | 19575 | 5.04 |  |
| *Aedes aegypti* | S | 4403 | 2.60 | 0.60 |
| *Aedes vexans* | L | 17779 | 2.78 |  |
| *Aedes vexans* | M | 75024 | 19.31 |  |
| *Aedes vexans* | S | 16826 | 9.95 | 1.50 |
| *Culex quinquefasciatus* | L | 355 | 0.06 |  |
| *Culex quinquefasciatus* | M | 394 | 0.10 |  |
| *Culex quinquefasciatus* | S | 231 | 0.14 | 0.02 |
|  |  |  |  |  |
|  |  |  |  |  |
| **S1 Table** |  |  |  |  |
