# Supplementary material for: RNA Interference Restricts Rift Valley Fever Virus in Multiple Insect Systems
Source: mSphere. 2017 May 3;2(3):e00090-17. doi: 10.1128/mSphere.00090-17 (PMC5415632; doi:10.1128/mSphere.00090-17)
Supplement: TABLE S2 [file sph002172276st4.docx]

| **Sample** | **Segment** | **genome 28nt** | **antigenome 28 nt** | **ratio (G:Ag)** | **total reads** | **reads per nt** | **% RVFV 28nt** |
| --- | --- | --- | --- | --- | --- | --- | --- |
| Aag2 cells | L | 13668 | 36690 | 2.68 | 50358 | 7.86 |  |
| Aag2 cells | M | 425857 | 243082 | 0.57 | 668939 | 172.14 |  |
| Aag2 cells | S | 174604 | 28535 | 0.16 | 203139 | 120.13 | 19.60 |
| *Aedes aegypti* | L | 1295 | 3412 | 2.63 | 4707 | 0.73 |  |
| *Aedes aegypti* | M | 20476 | 9625 | 0.47 | 30101 | 7.75 |  |
| *Aedes aegypti* | S | 12333 | 3659 | 0.30 | 15992 | 9.46 | 0.50 |
| *Aedes vexans* | L | 928 | 3121 | 3.36 | 4049 | 0.63 |  |
| *Aedes vexans* | M | 17570 | 17357 | 0.99 | 34927 | 8.99 |  |
| *Aedes vexans* | S | 10296 | 4328 | 0.42 | 14624 | 8.65 | 0.50 |
| *Culex quinquefasciatus* | L | 10 | 83 | 8.30 | 93 | 0.01 |  |
| *Culex quinquefasciatus* | M | 86 | 111 | 1.29 | 197 | 0.05 |  |
| *Culex quinquefasciatus* | S | 107 | 53 | 0.50 | 160 | 0.09 | 0.005 |
|  |  |  |  |  |  |  |  |
| **S2 Table** |  |  |  |  |  |  |  |
|  |  |  |  |  |  |  |  |
