# Supplementary figures and images for: RNA Interference Restricts Rift Valley Fever Virus in Multiple Insect Systems
Source: mSphere. 2017 May 3;2(3):e00090-17. doi: 10.1128/mSphere.00090-17 (PMC5415632; doi:10.1128/mSphere.00090-17)

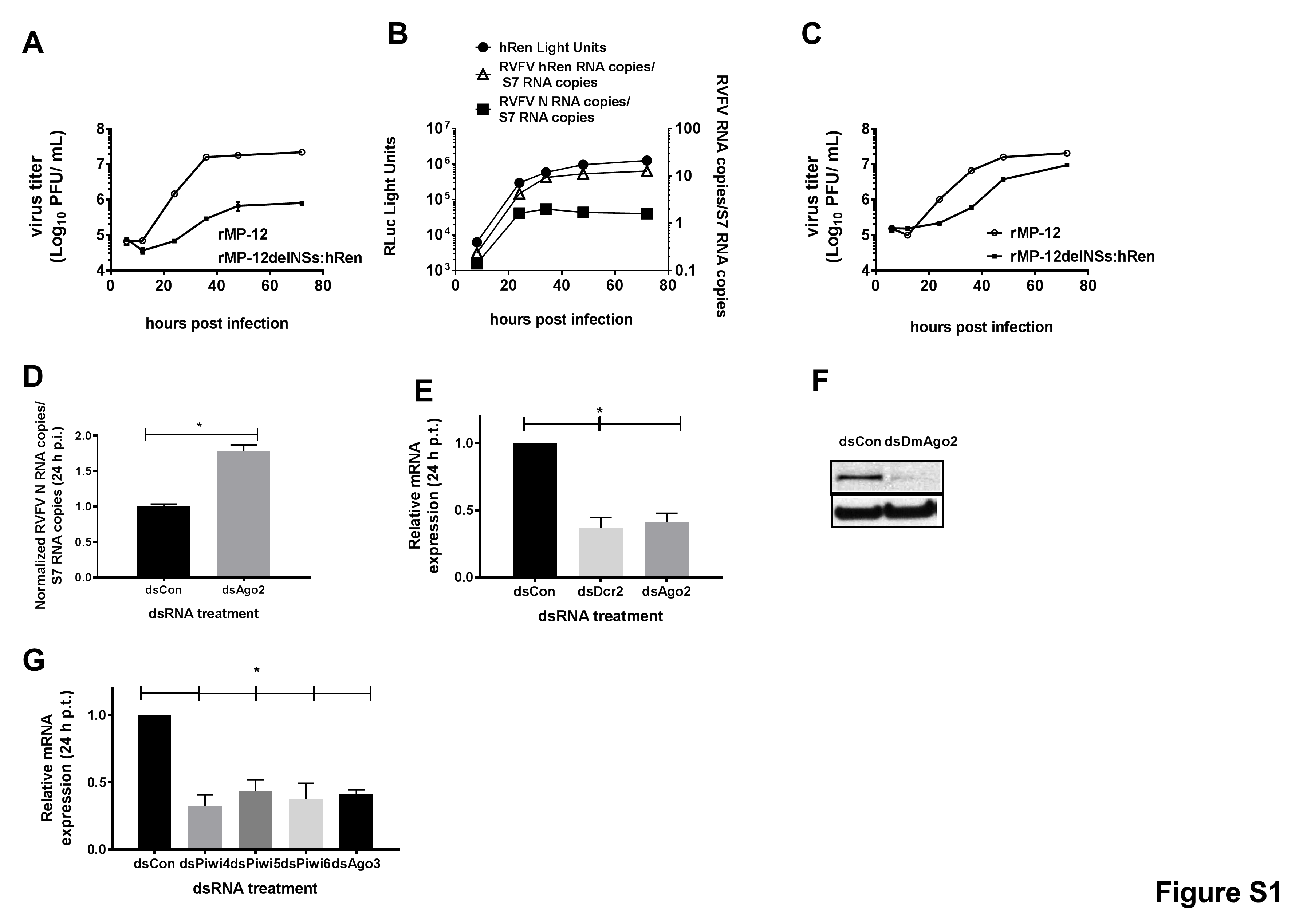

Supplement: FIG S1 [file sph002172276sf1.tif]

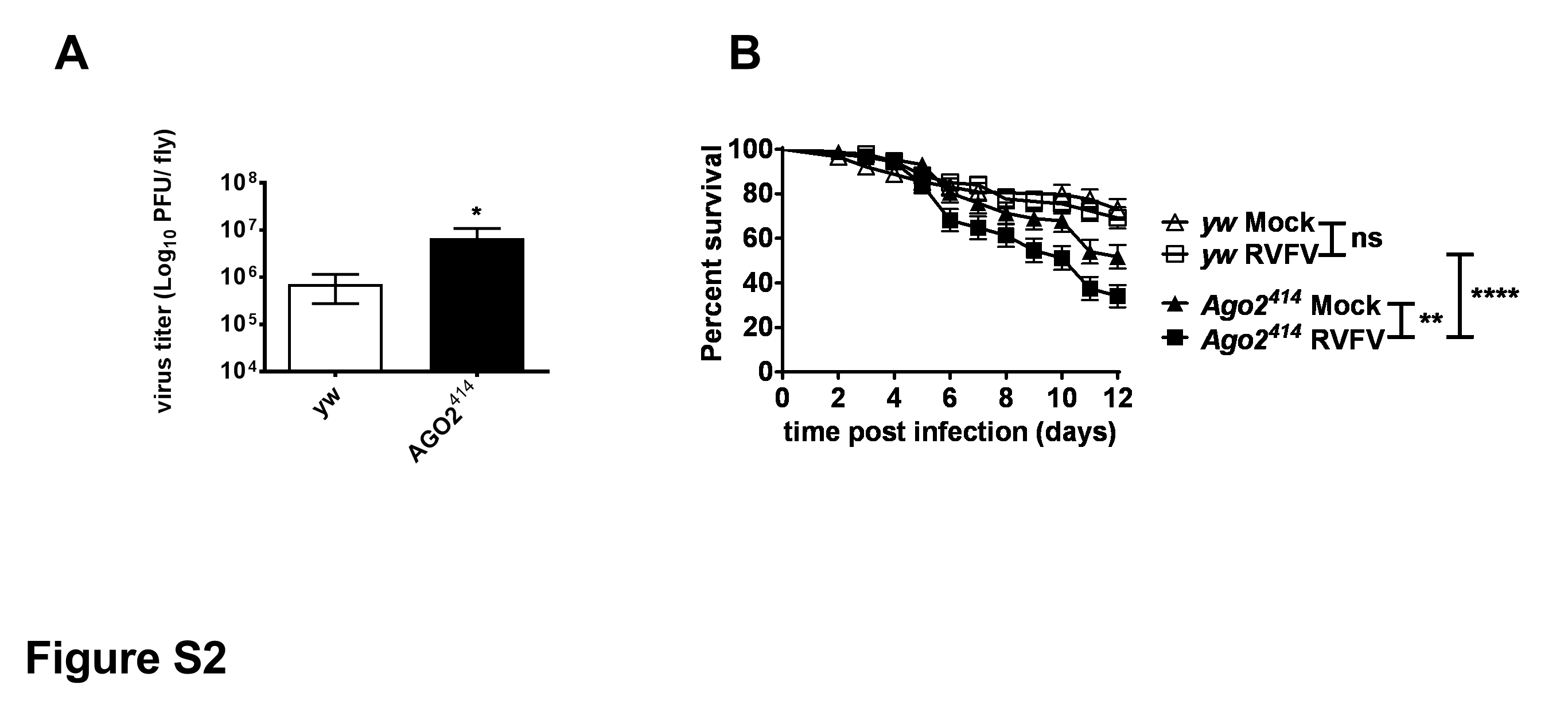

Supplement: FIG S2 [file sph002172276sf2.tif]
