# Supplementary material for: RNA Interference Restricts Rift Valley Fever Virus in Multiple Insect Systems
Source: mSphere. 2017 May 3;2(3):e00090-17. doi: 10.1128/mSphere.00090-17 (PMC5415632; doi:10.1128/mSphere.00090-17)
Supplement: TABLE S3 [file sph002172276st5.doc]

Table S3. Oligonucleotides used for PCR, cloning and qRT-PCR.

| Application | Primer name | Sequence (5`-3`) | Gene accession number | Gene position |
| --- | --- | --- | --- | --- |
| Reporter virus construction | forward | acaggaaagtggtacctgatacacgtgataagcactag |  |  |
|  | reverse | gaggaggagaggtaccatgatggactagttgaggttgattag |  |  |
| Reporter virus characterization | hRen FP | cgaacgcaaacgcatgatcactg | commercial vector phRL-CMV (Promega) |  |
|  | hRen RP | agaaaaatcacggcgttctcggc | commercial vector phRL-CMV (Promega) |  |
|  | hRen Probe | FAM-ctgcaagcaaatgaacgtgctggactccttcatcaa-TAM | commercial vector phRL-CMV (Promega) |  |
|  | RVF FP | tgccacgagtyagagcca | gb|DQ380154.1 | 1457-1474 |
|  | RVF RP | gtgggtccgagagtytgc | gb|DQ380154.1 | 1567-1584 |
|  | RVF Probe | FAM-tccttctcccagtcagccccac-BHQ1 | gb|DQ380154.1 | 1495-1515 |
|  | Aag-S7 FP | ccagcagaccaccattgaacacaag | gi|157123488 | 551-575 |
|  | Aag-S7 RP | ccgggaattcgaacgtaacgtcac | gi|157123488 | 616-639 |
|  | Aag-S7-Probe | FAM-ccggtcagcttcttgtacactgacgtgaaggt-TAM | gi|157123488 | 582-613 (binds antisense) |
| Silencing of RVFV using dsRNAs | dsL-Fwd | gtaatacgactcactatagggcaagtcaatatatcagatgaggg | gb|DQ375404.1 | 5640-5660 |
|  | dsL-Rev | gtaatacgactcactatagggagaactttcctaactgaggctctc | gb|DQ375404.1 | 6132-6155 |
|  | dsM-Fwd | gtaatacgactcactataggggttcacatgctaatgggtcag | gb|DQ380208.1 | 1321-1341 |
|  | dsM-Rev | gtaatacgactcactatagggcaggcacttaagcaccctataaag | gb|DQ380208.1 | 1827-1850 |
|  | dsN-Fwd | gtaatacgactcactatagggtgcccgtagagttatcgaactc | gb|DQ380154.1 | 1533-1554 |
|  | dsN-Rev | gtaatacgactcactatagggtgctattcactgctgcattcattg | gb|DQ380154.1 | 1030-1053 |
|  | dshRen-Fwd | gtaatacgactcactataggggtgctggactccttcatcaactac | commercial vector pGL4.75 (Promega) |  |
|  | dshRen-Rev | gtaatacgactcactataggggcatggtctcgacgaagaagttat | commercial vector pGL4.75 (Promega) |  |
| Small RNA sensor assays | Nanoluc-Fwd | actgaagcttatggtcttcacactcgaagatttcg | commercial vector pNL1.1(Promega) |  |
|  | Nanoluc-Rev | acatctagattacgccagaatgcgttcgcacagc | commercial vector pNL1.1(Promega) |  |
|  | RVFV L F XbaI | actgtctagacaagtcaatatcagatgagg | gb|DQ375404.1 | 5640-5659 |
|  | RVFV L R SacII | acaccgcggagaactttcctaactgaggctc | gb|DQ375404.1 | 6134-6155 |
|  | RVFV M Fwd XbaI | actgtctagagttcacatgctaatgggtca | gb|DQ380208.1 | 1321-1340 |
|  | RVFV M Rev SacII | acaccgcggcaggcacttaagcaccctat | gb|DQ380208.1 | 1831-1850 |
|  | RVFV N Fwd XbaI | actgtctagagtgcccgtagagttatcgaac | gb|DQ380154.1 | 1532-1552 |
|  | RVFV N Rev SacII | acaccgcggtgctattcactgctgcattc | gb|DQ380154.1 | 1034-1053 |
|  | NSs5’ F XbaI | actgtctagaatggattactttcctgtgatatc | gb|DQ380154.1 | 35-57 |
|  | NSs5’ R SacI | acaccgcggatcccttctaatgtcatcattc | gb|DQ380154.1 | 430-451 |
|  | NSs3’ F XbaI | actgtctagagactcctttgctggcttacac | gb|DQ380154.1 | 503-523 |
|  | NSs3’ R SacII | acaccgcggctaatcaacctcaacaaatccatc | gb|DQ380154.1 | 809-832 |
|  | eGFP-XbaI-FW | cctctagaggggcgtgcagtgcttcagccgc | commercial vector pEGFP-C1 (Clontech) |  |
|  | eGFP-SacII-RV | ccgcgggtggttgtcgggcagcagcac | commercial vector pEGFP-C1 (Clontech) |  |
| RVFV L-Segment PCR for virus quantification in *Drosophila* | RVFV-F | tgaaaattcctgaaacacatgg | gb|DQ375404.1 | 2912-2933 |
|  | RVFL-R | acttccttgcatcatctgatg | gb|DQ375404.1 | 3001-2981 |
|  | RVFL-Probe | FAM-caatgtaaggggcctgtgtggacttgtg-BHQ1 | gb|DQ375404.1 | 2950-2977 |
|  | RpL32 | commercial assay | Dm02151827_g1 |  |
|  | CG13220 | commercial assay | Dm01819575_s1 |  |
